# Methylation multiplicity and its clinical values in cancer

**DOI:** 10.1017/erm.2021.4

**Published:** 2021-03-31

**Authors:** Xiaofeng Dai, Tiejun Ren, Yuxin Zhang, Nan Nan

**Affiliations:** 1 Wuxi School of Medicine, Jiangnan University, Wuxi, Jiangsu 214122, China; 2 The Affiliated Luoyang Central Hospital of Zhengzhou University, Zhengzhou University, Luoyang, Henan 471000, China

**Keywords:** Cancer, DNA methylation, early diagnosis, protein methylation, RNA methylation

## Abstract

Methylation at DNA, RNA and protein levels plays critical roles in many cellular processes and is associated with diverse differentiation events, physiological activities and human diseases. To aid in the diagnostic and therapeutic design for cancer treatment utilising methylation, this review provides a boutique yet comprehensive overview on methylation at different levels including the mechanisms, cross-talking and clinical implications with a particular focus on cancers. We conclude that DNA methylation is the sole type of methylation that has been largely translated into clinics and used for, mostly, early diagnosis. Translating the onco-therapeutic and prognostic values of RNA and protein methylations into clinical use deserves intensive efforts. Simultaneous examination of methylations at multiple levels or together with other forms of molecular markers represents an interesting research direction with profound clinical translational potential.

## Introduction

Methylation represents an important type of epigenetic regulation that refers to the transfer of active methyl group to the target chemicals as catalysed by methyltransferases without altering the DNA sequence composition. Methylation does occur in DNA (Ref. 1), RNA (Ref. 2), histone (Ref. 3) and non-histone (Ref. 4) proteins. The dynamics and biological outcomes of all three types of methylation are the results of the activity of a complex protein machinery comprised of writers, erasers and readers. Methylation deregulation is involved in many diseases including human cancers (Refs 5–7). DNA methylation transcriptionally modulates the expression of the target gene (Ref. 8); RNA methylation primarily modulates RNA processing and decay (Ref. 9); protein methylation affects protein activities and directs protein translation, localisation and signalling (Ref. 10). These methylation events play broad roles in regulating cell fates, and their cross-talks as well as that with other post-translational modification (PTM) events exert a reversible control over various cellular behaviours (Ref. 11).

This review will introduce the primary enzymes and mechanism that enable each type of methylation, followed by a brief discussion on the cross-talks among methylation at multiple levels. This paper is finalised with clinical implications of methylation, with the aim of contributing to the clinical translation of methylation-related knowledge from the bench side to the bedside.

## Methylation types

Methylation is a reversible switch controlled by specific catalytic enzymes, that is, methyltransferase (writer), demethylase (eraser) and function through being recognised by methylation-dependent binding protein (reader). DNA methylation typically occurs as 5-methylcytosine (m5C), with DNA methyltransferase 1 (DNMT1) being the key methyltransferase and the TET family proteins playing the primary demethylation role. The most representative RNA methylations are N6-methyladenosine (m6A) and m5C, where m6A is catalysed by the methyltransferase-like 3/14 (METTL3/14), Wilms tumour 1-associated protein (WTAP) methyltransferase complex (METTL3/METTL4/WTAP) and removed by obesity-associated enzyme (FTO) and alkylation repair homologue 5 (ALKBH5), and m5C is methylated by NOP2/Sun RNA methyltransferase (NSUN) family proteins and DNA methyltransferase family members as represented by DNMT2 with the demethylases being unclear. Protein methylation can occur on both histone and non-histone proteins, and typically at arginine and lysine. The most frequently reported histone methylations are H3K, H4K, H3R and H4R. Non-histone protein methylation is typically involved in signal transduction, many of which are associated with cancer progression such as MAPK and NFkB signalling. Arginine methylation is triggered by protein arginine methyltransferase (PRMT) family proteins, and lysine methylation is mediated by lysine methyltransferase (KMT) that contains a highly evolutionarily conserved SET domain. Jumonji C-terminal domain (JmjC) family members and lysine-specific demethylase 1 (LSD1) are the primary demethylases unveiled for lysine demethylation, and some members of the JmjC family such as KDM3A, KDM4E, KDM5C are known to catalyse arginine demethylation as well.

### DNA methylation

DNA methylation is an epigenetic process that adds a methyl (CH_3_) group to DNA using S-adenosyl methionine (SAM) as the methyl group donor. This process can occur at N6 of adenine, N7 of guanine, N4 and C5 of cytosine. Approximately 80% of cytosine-phosphate-guanine (CpG) dinucleotides are methylated in mammalian cells, which typically occurs in the 5′ end of cytosine by converting C5 of cytosine to m5C (Refs 12, 13).

#### Writer: DNA methyltransferase

**Fig. 1.**
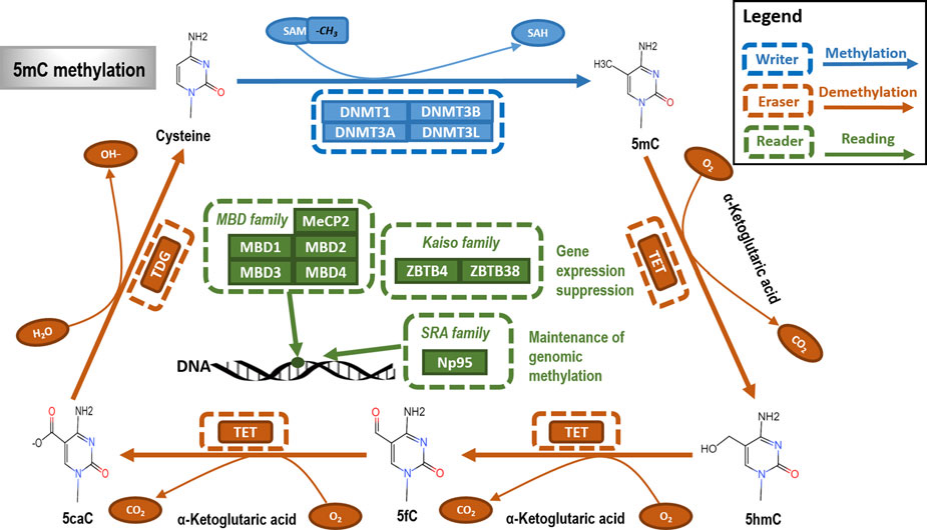
The primary type and mechanism of DNA methylation. DNA methylation typically occurs as 5mC, with DNMT1 being the key methyltransferase (writer) and the TET family proteins playing the primary demethylation (eraser) role. Three kinds of DNA methylation-binding proteins (reader) were reported, which are MBD (Methyl-CpG-Binding Domain), Kaiso and SRA (Set and Ring Finger-associated) families. Readers identify methylated DNA sites to enable the downstream effects including, for example, gene expression suppression and genomic methylation maintenance.

DNMT family proteins are primarily comprised of DNMT1, DNMT3A, DNMT3B and DNMT3L (Fig. 1). DNMT1 is the key enzyme that maintains the normal DNA methylation level during DNA replication (Ref. 14). DNMT3A and NDMT3B are highly homogeneous that catalyse *de novo* methylation in early and late embryonic processes, respectively (Ref. 15). DNMT3L, though without the methyltransferase activity, facilitates DNMT3A/B in exerting their functionalities (Ref. 16).

#### Eraser: DNA demethylase

Strictly speaking, no enzyme in humans could be called ‘DNA demethylase’. The TET family members, belonging to an evolutionarily conserved family of dioxygenases, play key regulatory roles during DNA demethylation (Fig. 1). On TET catalysis, m5C is hydroxylated into 5-hydroxymethyl cytosine (5hmC) followed by further oxidisation into 5-formylcytosine and 5-carboxylcytosine. Thymine DNA glycosylase further converts m5C into cytosine through the base-excision repair pathway (Ref. 14).

#### Reader: DNA methylation-dependent binding protein

There are, so far, three kinds of DNA methylation-binding proteins reported, which are MBD (Methyl-CpG-Binding Domain), Kaiso and SRA (Set and Ring Finger-associated) families (Fig. 1). The MBD family is comprised of MeCP2, MBD1, MBD2, MBD3 and MBD4 in mammals, four of which, that is, MeCP2, MBD1, MBD2 and MBD4 bind a symmetrically methylated CpG motif (Refs 17, 18). Kaiso family members ZBTB4 and ZBTB38 transcriptionally suppress gene expression via binding methylated DNA through zinc finger proteins in vivo (Ref. 19). The SRA family member Np95 protein recruits DNMT1 to hemi-methylated CpG loci for the maintenance of genomic methylation (Ref. 20).

### RNA methylation

RNA methylation is a type of chemical modification that occurs in various types of RNAs such as messenger RNA (mRNA), transfer RNA (tRNA), ribosome RNA and small nuclear RNA. Over 150 types of RNA methylation have been discovered, including m6A, m5C, 1-methyladenosine, 5-hydroxymethylcytosine, with m6A and m5C being the most representative and intensively studied RNA methylation types. Below we will introduce the primary components required in RNA methylation and how they orchestrate to determine the RNA methylation process using m6A and m5C as the examples.

#### Writer: RNA methyltransferase

**Fig. 2.**
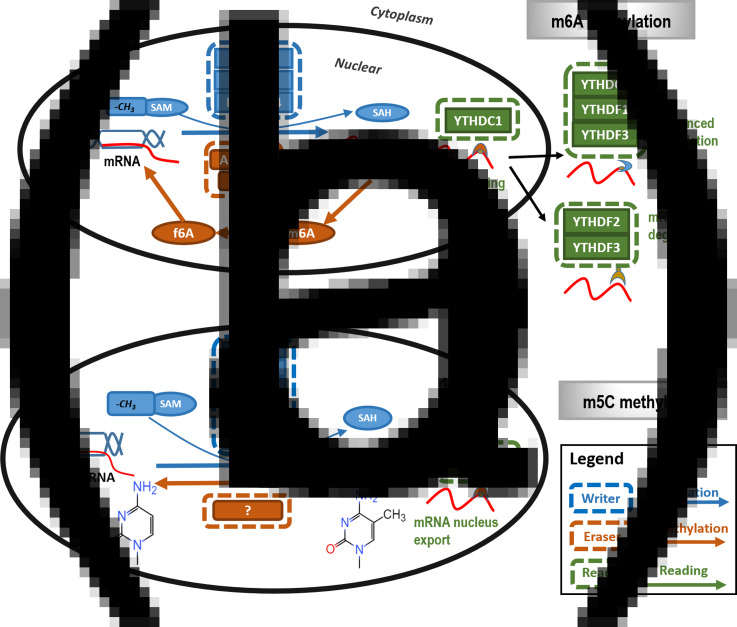
The primary types and mechanisms of RNA methylation. The most representative RNA methylations are m6A and m5C. (a) In m6A RNA methylation, m6A is catalysed by the METTL3/METTL14/WTAP methyltransferase complex (m6A writer) and removed by FTO and ALKBH5 (m6A eraser). In addition, YTHDC1, YTHDC2 and YTHDF1 to YTHDF3 are m6A methylation-dependent binding proteins (m6A reader) to enable functionalities such as regulating mRNA splicing, enhance translation and mediate mRNA degradation. (b) In m5C RNA methylation, m5C is methylated by NSUN family proteins and DNMT family members as represented by DNMT2 (m5C writer) with the demethylases (m5C eraser) being unclear. ALYREF is so far the only recognised m5C methylation-dependent binding protein (m5C reader) that functions in availing mRNA nucleus export.

The m6A RNA methylation process, specifically present in eukaryotes and accounting for over 80% of all RNA methylation, is catalysed by the RNA methyltransferase complex using SAM as the methyl group donor. The core of the m6A methyltransferase complex is primarily composed of METTL3, METTL14 and WTAP (Fig. 2a). METTL3 is the first RNA methyltransferase being discovered that plays fundamental catalytic roles in m6A methylation. METTL14 could enhance the catalytic activity of METTL3 via forming heterodimers with METTL3 with the ratio of 1:1 (Refs 21, 22). WTAP could guide and localise the m6A methyltransferase complex on the nucleosome of mRNA targets to exert the catalytic functionalities (Ref. 23). Besides the core components, the m6A methyltransferase complex also contains other proteins such as virus-like m6A methyltransferase associated that could recruit the core components to specific locations for methylation (Ref. 24), and zinc finger CCCH-type containing 13 that could promote the nucleus localisation of the core complex (Ref. 25).

The m5C methyltransferase complex is composed of NSUN and DNMT2 family proteins, which catalyses the transfer of the methyl group from the SAM donor to cytosines of the target RNAs (Ref. 26). The NSUN family has nine members, where NSUN1, NSUN2, NSUN3, NSUN4, NSUN5 are known with catalytic activities on m5C demethylation (Fig. 2b). The catalytic role of NSUN2 is most clearly elucidated that depends on two cysteine sites, C321 and C271. Although C321 catalyses the methylation process of cytosines, C271 mediates the release of RNA after methylation (Ref. 27). DNMT2 could catalyse the methylation of miRNA and C38 sites of tRNAs of aspartic acids (Ref. 28).

#### Eraser: RNA demethylase

The RNA methylation process could be reversed by RNA demethylases. Typical m6A RNA demethylases include FTO and ALKBH5 which both belong to the ALKB protein family and take on the catalytic role in a Fe^2+^ and *α*-ketoglutaric acid-dependent manner (Ref. 29). FTO is the first discovered RNA demethylase that demethylates m6A via an intermediate step that oxidises m6A to N6-hydroxymethyladenosine and N6-formyladenosine (Fig. 2a). Unlike FTO, ALKBH5 could directly demethylate m6A into adenine without generating intermediate products (Ref. 30). Relatively little has been reported on m5C demethylases (Fig. 2b).

#### Reader: RNA methylation-dependent binding protein

The m6A binding proteins bind m6A to help it exert biological functionalities. Proteins harbouring the YTH domain, a domain that is present in close to 200 different proteins and evolutionarily conserved across the eukaryotic species, are the primary m6A binding proteins which include YTHDC1 to YTHDC2 and YTHDF1 to YTHDF3 (Ref. 31) (Fig. 2a). YTHDC1, a core member of the YTH family, is localised in the nucleus and could regulate mRNA splicing by selectively recruiting splicing factors to the target sites (Ref. 32). YTHDC2 contains multiple DNA helicase domains that can bind m6A sites to enhance its translation efficacy (Ref. 33). YTHDF1 could enhance the translation efficiency of mRNA via interacting with ribosome and transcription factors (Ref. 34). YTHDF2 could accelerate the degradation of m6A modified transcripts (Ref. 35). YTHDF3 could interact with YTHDF1 or YTHDF2 to enhance its ability in enhancing translation and mediating protein decay (Refs 36, 37).

ALYREF is so far the only recognised m5C methylation-dependent binding protein (Fig. 2b). Silencing and over-expressing *ALYREF* could increase and decrease nucleus mRNA expression, respectively, which could not be achieved by modulating the expression of *ALYREF* that is deficient in m5C binding using fluorescence *in situ* hybridisation, implicating that ALYREF could help regulate the nucleus export process of mRNA through binding the m5C site of mRNA (Ref. 27).

### Protein methylation

**Fig. 3.**
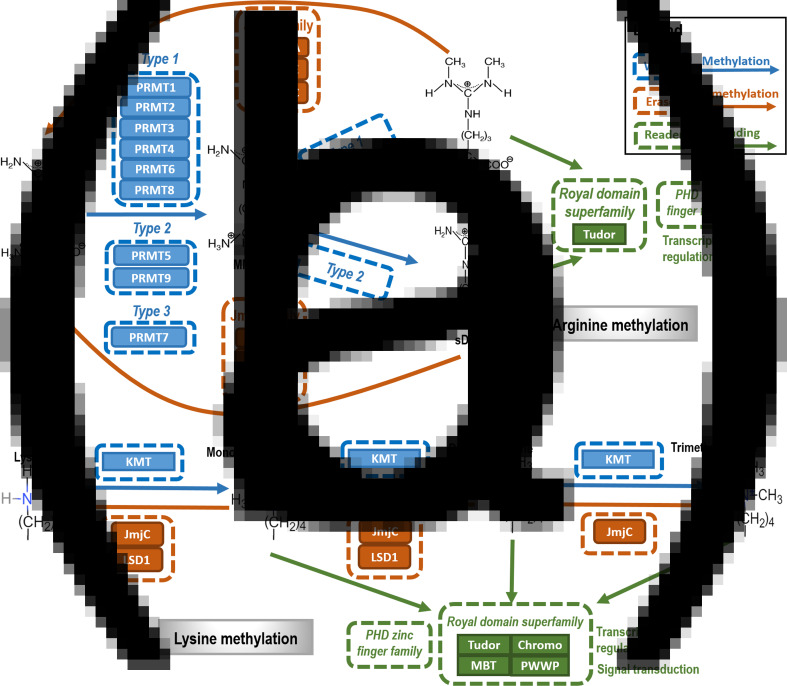
The primary types and mechanisms of protein methylation. Protein methylation can occur on both histone and non-histone proteins, and typically at arginine and lysine. (a) Arginine methylation is triggered by PRMT (Arg methylation writer). Some members of the JmjC family such as KDM3A, KDM4E, KDM5C (Arg methylation eraser) are known to catalyse arginine demethylation. The Tudor domain and PHD zinc finger domain recognise arginine methylation (Arg methylation reader) to play diverse roles in many cellular processes. (b) Lysine methylation is mediated by lysine methyltransferase, abbreviated as KMT (Lys methylation writer). JmjC family members and LSD1 are the primary demethylases unveiled for lysine demethylation. The ‘royal’ domain superfamily, comprised of Tudor, chromo, MBT and PWWP domains, and PHD zinc finger domain are lysine methylation readers.

Post-transcriptional modifications of the core histones (i.e. H2A, H2B, H3, H4) including methylation are considered as the ‘histone code’ that prime gene expression among other functionalities (Refs 38–44). Histone methylation is the most intensively studied type of histone modifications that typically occurs in the arginine and lysine arginine residues of histone 3 (H3) and histone 4 (H4) (Ref. 11). Arginine may be mono- or dimethylated on its side chain (Ref. 45), and the *ε*-amino group of lysine may be mono-, di- or trimethylated (Refs 46–48) (Fig. 3). Besides histones, protein methylation can also occur on non-histone proteins at arginine and lysine residuals, which has become a prevalent PTM and an important regulator of cellular signal transduction as mediated by, for example, MAPK, WNT, BMP, Hippo, JAK-STAT, p53 and NFkB signalling (Refs 11, 49–51). There also exists some less frequent methylation at other amino acid residues such as serine (Ref. 52).

#### Writer: protein methyltransferase

Arginine methylation is triggered by PRMT that catalyses the transfer of the methyl group from SAM to nitrogen atom (N) of the guanidine group of arginine (Ref. 48). There are nine types of PRMT discovered in mammals that could be classified into three types. Type I PRMT is comprised of PRMT1, PRMT2, PRMT3, PRMT4, PRMT6 and PRMT8, which catalyse arginine into mono-methylarginine (MMA) and asymmetric dimethylarginine; type II PRMT includes PRMT5 and PRMT9 that catalyse arginine into MMA and symmetric dimethylarginine; and type III PRMT refers to PRMT7 that catalyses only the formation of MMA(Ref. 53) (Fig. 3a).

Lysine methylation is mediated by KMT that can trigger mono-, di- and triple-methylation of lysine (Ref. 48) (Fig. 3b). Most KMTs contain the SET domain that is highly evolutionarily conserved. The SET domain forms the methylation complex with the aid of some structural subunits which are comprised of the pre-SET and post-SET domains. Both ends of the SET domain are fundamental to the maintenance of KMT activity, with the N terminal being the region for interactions and the C terminal being critical to the catalytic activity of KMTs (Ref. 54).

#### Eraser: protein demethylase

Not until 2004 when the first protein demethylase was discovered, protein methylation has been considered a non-reversible process ever since its first discovery (Ref. 55). There are two classes of histone lysine demethylases, which are the amine oxidase-related enzymes as represented by LSD1 and the JmjC family of demethylases (Fig. 3b). LSD1 is the first discovered histone demethylase, which is a flavin adenine dinucleotide-dependent enzyme catalysing the reduction of the mono- and di-methylation of histone lysine. LSD1 is composed of three domains, that is, the Tower, SWIRM and amine oxidase domains. The Tower domain is located in the structural centre, the loss of which is associated with the lack of enzyme activity. The SWIRM domain is located in the N terminal and is a protein–protein interaction motif. The amine oxidase domain harbours the catalytic activity and is located in the C terminal. The JmjC family of demethylases are Fe^2+^ and *α*-ketoglutarate-dependent dioxygenase that catalyse the reduction of mono-, di- and triple methylation processes of histone lysine. The JmjC domain located in the N and C terminals of JmjD family members is the core component of the enzyme activity centre (Ref. 39). Among these lysine demethylases, only four are known to act on non-histone proteins, which are LSD1, KDM4A, KDM2A and PHF2 (Ref. 56).

The existence of arginine demethylases has once been controversial (Ref. 57). Yet it was demonstrated in the recent 5 years that certain lysine demethylases such as KDM3A, KDM4E and KDM5C are also erasers of methylated arginine (Ref. 58) (Fig. 3a). Given the similar demethylation process of arginine with lysine, other JmjC family proteins may also play a demethylation role on methylated arginine (Ref. 53).

#### Reader: protein methylation-dependent binding protein

The ‘royal’ domain superfamily typically recognises arginine- or lysine-methylated regions and is comprised of Tudor, chromo, MBT and PWWP domains. Although the chromo, MBT and PWWP domains could specifically identify methylated lysine, the Tudor domain could recognise both lysine and arginine methylation (Ref. 59) (Fig. 3). PHD zinc finger domain is another family of readers besides the ‘royal family’ that was identified in 1993. The PHD finger harbours the ‘Cys4HisCys3’ zinc finger motif and could bind multiple types of methylated residues in histone or non-histone proteins to play diverse roles in many cellular processes including transcriptional regulation and signal transduction (Ref. 60).

## Methylation cross-talk

With advances in the development of these detection approaches and the increasing number of methylation loci as well as their cellular functionalities, cross-talking among methylations of the same type or across different types or even with other types of PTM events was discovered, suggesting the complexity of methylation and the delicate nature of epigenetic regulation in orchestrating the complicated machinery of human life.

### Cross-talk between methylation types

Interplays exist between DNA and histone methylations. Accumulating evidence has suggested that some *de novo* DNA methylation relies on pre-existing histone lysine methylation and *vice versa*. For instance, H3K9 methylation is critical in maintaining DNA methylation, and H3K9me3/me2 levels decrease at heterochromatic regions where DNA methylation is lost that typically occurs in cancer cells (Ref. 61). It was also reported that the direct association of DNMTs with H3K9 methyltransferases might play an essential role in targeting *de novo* DNA methylation with the exact mechanism unclear (Ref. 62). Similar roles have also been observed between H3K36 and *de novo* DNA methylation. The enrichments of H3K36 methylation and DNA methylation are positively correlated in gene bodies (Refs 63–67), which seems to synergistically regulate the splicing machinery (Ref. 68). This suggests that H3K36 methylation may help recruit DNMT3a/b to gene bodies for successful DNA methylation. Besides increasing DNA methylation, there are also facts suggesting that histone methylation may play the opposite roles. For example, H3K4me3 seems to be mutually exclusive with *de novo* DNA methylation (Ref. 69), which may be because of the blockage of the interaction between H3K4 and DNMT3-associated protein DNMT3L (occurring through the ADD domain of DNMT3L) by H3K4 methylation (Ref. 70). The cross-talk between DNA and histone methylation also occurs in other histone sites such as H3K27me3 that has associations of both directions and H3K9me2 that is protective of DNA from demethylation. For instance, it was reported that H3K27me3 extensively overlapped with DNA methylation in somatic and cancer cells, but located in discrete regions generally devoid of DNA methylation in embryonic stem cells (Refs 71, 72). It was demonstrated that the maternal genome, following fertilisation, and certain paternal imprinted loci are protected from TET3-mediated hydroxylation (m5C to 5hmC) through binding PGC7 to H3K9me2 (Ref. 73).

As histone methylation plays critical roles in gene transcription that is the endpoint of cellular signal transduction as mediated partially via non-histone protein methylation, there must exist a cross-talk between histone and non-histone methylations. Thereby, intensively efforts on how histone methylation is integrated with non-histone protein methylation in the regulation of cellular and nuclear processes such as DNA damage repair are needed. An example here is the interplay between NUMB and p53 that is modulated by methylation, where NUMB is an endocytic adaptor protein capable of binding p53 and thus preventing it from ubiquitylation by MDM2 (Ref. 74). In normal condition, NUMB is methylated by SETD8 on Lys158 and Lys163 and thus loses the ability of binding p53, which leads to enhanced p53 decay (Ref. 75). In response to DNA damage and H4K20 mono-methylation, SETD8 is ubiquitylated by CRL4 that results in NUMB demethylation, the formation of the NUMB-p53 complex, reduced p53 degradation and increased p53 activity (Ref. 76). Thus, the cross-talk between histone and non-histone protein methylations here dictates p53 activity and thus constitutes a checkpoint that determines cell fate during DDR.

### Cross-talk with other PTMs

Cross-talk of protein methylation can be found with methylation residues (Ref. 77). One typical example is p53, which could be methylated at multiple arginine sites (Are 333, Arg 335, Arg 337) by PRMT5 and at multiple lysine sites (Lys 370, Lys 372, Lys 373, Lys 382). P53 mono- and di-methylation at Lys 370 by SMYD2 repress and enhance its transcription, respectively (Refs 78–80), and both mono- and di-methylation of Lys 370 are inhibited by the di-methylation of the neighbouring Lys 372 by SETD7 (Ref. 79). Thus, p53 expression and functionalities are orchestrated by the dynamic methylation of these two nearby residues, Lys 370 and Lys 372 (Ref. 79).

Cross-talk of methylation occurs not only between neighbouring methylation sites but also occur with other PTMs such as phosphorylation (Ref. 81). For instance, arginine methylation could increase the stability and activity of FOXO1 by inhibiting its phosphorylation. Specifically, FOXO1 phosphorylation on Ser253 by AKT leads to its cytoplasmic retention, ubiquitylation and subsequent degradation, whereas FOXO1 methylation at residues Arg248 and Arg250 by PRMT1 blocks AKT-dependent phosphorylation (Ref. 82). Direct interaction with neighbouring phosphorylation sites is called the ‘methylation–phosphorylation switch’ (Ref. 81). One example is the switch in DNMT between its methylation at lysine 142 by SETD8 and its phosphorylation at serine 143 by AKT (Ref. 83), which are mutually exclusive and collectively orchestrate the activation status of DNMT (i.e. phosphorylated DNMT is active, methylated DNMT is inactive) (Ref. 83). Similar switches also exist in other proteins such as the cell cycle regulator RB (Ref. 84) and the transcription factor NFkB (Ref. 85).

### Cross-use of methylation enzymes

Though the mechanisms differ, methylation at different levels may share the same enzymes in different organisms. For instance, the SPOUT family of RNA methyltransferases has been found to methylate arginine in yeast (Ref. 86).

## Clinical relevance of methylation in cancers

### DNA methylation and cancer

In 1948, Rollin Hotchkiss separated an unknown material, the absorption characteristics resemble those of cytosine using paper chromatography (Ref. 87), and Gerard Wyatt confirmed its identity as m5C in the 1950s (Refs 88, 89). In 1975, Holliday and Pugh provided the first evidence on the regulatory role of DNA methylation in gene expression (Ref. 90), opening the avenue of exploring the disease association of DNA methylation.

CpG island DNA methylation is rare in normal cells and increases with age (Ref. 91). It plays critical roles in X-chromosome inactivation (Ref. 92), imprinting (Ref. 93) and cancers (Ref. 94). Accumulating evidence has suggested the existence of CpG island promoter hypermethylation of tumour suppressor genes such as *BRCA1* and *FOXO3a* in breast cancer (Refs 94, 95), *SET9* in cervical cancer (Ref. 96), *pRB* in familial cases of unilateral retinoblastoma (Ref. 97), *VHL* in renal carcinoma (Ref. 98), *p16INK4a* in melanoma (Ref. 99), *p15INK4b* in hematologic malignancies (Ref. 100), *hMLH1* and *APC* in sporadic colorectal cancers (Refs 101, 102). DNA methylation could also occur on oncogenes. It was reported that the promoter region of the oncogene *KIT* was methylated in over 43/110 melanoma cell lines, 3/12 primary and 11/29 metastatic cutaneous melanomas; however, the mechanism underlying the tumour suppressive role of this methylation event remains unclear (Ref. 103). In addition, the oncogene *MUC1* is frequently over-expressed in malignant tissues, the expression of which is enhanced and thus promotes cancer stemness as a result of DNMT1-mediated methylation (Ref. 104). These associations may convey profound clinical implications. For instance, hyper-methylated SETP9 exhibited a high sensitivity and specificity in cervical cancer diagnosis (Ref. 96), and the critical role of the DNMT1/FOXO3a/FOXM1/SOX2 pathway in regulating breast cancer stemness has been suggested as a potential therapeutic target for breast cancer treatment (Ref. 94). Several studies also revealed the potential direct regulatory functionality of DNA methylation on tumourigenesis. For instance, treating colon cells containing a hypermethylated *hMLH1* gene with the DNMT1 inhibitor 5-aza-2′-deoxycytidine restored *hMLH1* expression, a mismatch repair (MMR) gene, and MMR activity, suggesting the driving role of *hMLH1* CpG island hypermethylation in the observed microsatellite instability that is caused by MMR deficiency in sporadic colorectal carcinomas (Ref. 102). It was also reported that the tumourigenesis of some cancers is caused by the hypermethylation of one copy of a tumour suppressor gene whereas the other copy is naturally mutated or lost, supporting the role of DNA hypermethylation in driving tumourigenesis (Ref. 105).

### RNA methylation and cancer

RNA methylation has an unprecedented impact on many critical cellular events such as tissue development, circadian rhythm regulation, DNA damage repair, as well as tumour initiation and progression through largely regulating RNA stability (Refs 106–108). The functionalities of RNA methylation as represented by RNA methyltransferases are double-edged in tumour initiation and progression. RNA methylation typically reduces the expression of the downstream targets, rendering its roles highly dependent on the nature of the target genes. For instance, the m6A methyltransferase METTL3 is over-expressed in gastric, colon and liver cancers and promotes cancer cell proliferation via suppressing the tumour suppressor SOCS2 (Refs 109–111). High level of METTL3 upregulates HBXIP expression that inhibits the tumour suppressor let-7g through m6A methylation, and the positive feedback loop HBXIP/let-7g/METTL3/HBXIP promotes cancer cell proliferation in breast tumours (Ref. 112). The m5C methyltransferase NSUN2 is over-expressed in multiple types of tumours such as breast and colorectal malignancies (Ref. 113). NSUN2 protein expression was found to be upregulated in 34% breast tumours through immunohistochemical analysis of tissue microarrays (Ref. 114), and a pan-cancer analysis revealed a positive correlation of NSUN2 expression with its DNA copy number, as well as a positive association of NSUN2 over-expression with poor clinical outcome among breast cancer patients (Ref. 115). Yet, how NSUN2 promotes breast cancer cell progression requires further studies. NSUN2 was reported to be over-expressed in colorectal cancers that plays pro-migratory functionalities via methylating the precursor pri-miR-125b2 to block its processing into miR-125b that silences the expression of oncogenes including *GAB2* (Refs 116, 117). On the other hand, RNA methylation could be tumour suppressive. For instance, METTL3 over-expression could significantly suppress the proliferation and migration of renal cell carcinoma via perturbating the epithelial-to-mesenchymal transition and PI3K-AKT-mTOR pathways, yet the downstream target was unidentified (Ref. 118). The m6A methylation and m5C methylation could create synergies in regulating tumour states. For example, NSUN2 triggered m5C methylation and METTL3/METTL14 catalysed m6A methylation could synergistically upregulate the expression of p21, an important tumour suppressor, in senescent cells on redox stimulation (Ref. 119).

### Protein methylation and cancer

In 1959, a decade or so later after DNA methylation was discovered, Richard P. Ambler found *ε*-N-methyl-lysine from hydrochloric acid hydrolysates of the flagellin, which is the first discovery of protein methylation in living cells (Ref. 120). Later, Kim and Paik demonstrated that methylated lysine could not be conjugated to tRNAs, suggesting that histone methylation is a post-translational event (Ref. 121). This had substantially advanced our understanding on the role of protein methylation in many diseases including cancers.

Lysine methylation could either activate or repress gene expression, depending on the location and degree of methylation (Refs 38, 41–44, 46). For instance, histone H3K9, H3K27 or H4K20 methylation typically suppresses gene expression, whereas genes marked with H3K4 and H3K36 methylation are, in general, activated (Ref. 42). Take pancreatic cancer as an example, as EZH2 could suppress the expression of miR-139-5p via upregulating H3K27me3, decreased level of EZH2 could induce miRNA-139-5p expression and thus halt pancreatic cancer development (Ref. 122). H3K27me3 immunohistochemistry was also suggested to be a useful adjunct in meningioma diagnosis, especially for patients with WHO grade II histology or between WHO grades I and II (Ref. 123). In addition, the over-expression of G9a (a methyltransferase of H3K27 methylation) increases both H3K9 and H3K27 methylation that leads to reduced E-cadherin expression and triggers epithelial–mesenchymal transition in PANC-1 pancreatic cancer cells (Ref. 124). On the other hand, H3K4me3 is enriched in the cd274 promoter that activates PD-L1 transcription in pancreatic cancer cells (Ref. 125).

Approximately 90% arginine methylation is methylated by PRMT1 that catalyses both histone and non-histone proteins. For instance, PRMT1 could catalyse histone H4K3 asymmetric dimethylation and activate the expression of downstream genes including those involved in the Wnt/*β*-catenin and Notch signalling, and thus promote cancer initiation and progression such as that in oesophageal squamous cell carcinoma (Ref. 126). On the other hand, PRMT1 could promote the expression of Gli1 and thus contribute to the progression of pancreatic cancers by methylating Gli1 at Arg597 (Ref. 127); in addition, PRMT1 could also methylate Arg378 of Axin to inhibit its degradation, a negative regulator of Wnt/*β*-catenin pathway, and thus suppress cancer progression (Ref. 128). It was recently reported that protein arginine methylation promotes therapeutic resistance in human pancreatic cancer. HSP70 methylation at the arginine residue R416 enhances its binding ability to BCL2, which leads to the stabilisation of BCL2 mRNA and consequently reduced cancer cell apoptosis and therapeutic resistance (Ref. 129).

## Future perspective

Methylation represents a set of reversible and complicated events under delicate control and plays fundamental roles in human life including cancer initiation and progression. Deregulation of enzymes catalysing these events are typically associated with various abnormalities, and epigenetic therapies specifically targeting certain epigenetically defined cohorts of patients may be clinically feasible. The earliest successful epigenetic therapy is 5-Azacytidine, which specifically suppresses DNA methylation, has been recommended as the first-line therapy for high-risk myelodysplastic syndrome treatment in clinics (Ref. 130), yet successful methylation therapies against cancers are lacking that urges us to devote intensive efforts in this research domain. Thus, intensive efforts are needed to explore more methyltransferases, demethylases, methylation-binding proteins as well as their working mechanisms to enhance our understandings on this epigenetic event for clinical translation. Methylation is advantageous in its reversibility that makes the associated therapeutics mild and more controllable on the side effects as compared with gene therapies, but is disadvantageous in the relatively slow efficacy that may take months to show any therapeutic effects. Relatively little advance had been made on cancer therapeutics utilising altered methylation profiles under pathological conditions except for INQOVI® (cedazuridine/decitabine) that is used for treating chronic myelomonocytic leukaemia and myelodysplastic syndromes. Therefore, strategies combining epigenetic therapies with other approaches for enhanced therapeutic efficacy are required, and may represent another important direction to follow.

## Concluding remarks

DNA, RNA and protein methylation play fundamental and diverse roles in many cellular processes such as gene transcription, RNA splicing and decay, protein degradation and signal transduction, and were shown to be of critical value in various physiological and pathological events such as embryonic development, imprinting, circadian rhythm regulation, and the initiation and progression of malignancies. The close associations of various types of methylation and human diseases make them excellent candidates for cancer management such as early detection and therapeutic design. Besides, the universally existed and complicated cross-talks among methylation sites as well as their prominent roles in orchestrating many critical cellular processes also suggest the feasibility of methylation in serving as a therapeutic target or diagnostic maker in clinics. The reversibility nature of methylation makes it a potential mild yet effective approach for disease treatment which may require the joint use of other therapeutics to enhance its efficacy. The relatively early stage that methylation controls as compared with genetic alteration in complex diseases such as cancer makes methylation an ideal biomarker for early diagnosis or therapeutic efficacy monitoring (×Table 1). Despite the immense possibility that methylation may contribute to the clinical control of cancer, relatively little success has been made in clinics that require intensive efforts. Importantly, the use of epigenetic modulation including methylation at various levels in clinics for complex disease control including cancers is case-dependent as there is no universal rule on what efficacy could be achieved by methylation/demethylation at a particular locus in a certain disease and warrants special attention. This makes methylation a key member of the next generation of precision medicine.

**Table 1.** Clinical advances in using methylation as diagnostic markers for cancer early detection

Product name	Target gene	Disease	Detection	Sample	Company	Status	Country
EarlyTect	SDC2	Colorectal cancer	qMSP	Stool	Genomictree	KFDA approved	Korea
Coloscape	2-gene DNA methylation (NDRG4, BMP3), 20 loci from 4-gene mutation (APC, KRAS, BRAF, CTNNB1)	Colorectal cancer	qMSP	Blood	Diacarta	Trademark registered	USA
Cologuard	2-gene DNA methylation (NDRG4, BMP3), 7 DNA point mutations of KRAS, *β*-actin, haemoglobin immunoassay	Colorectal cancer	qMSP	Stool	Exact Sciences	FDA approved	USA
Epi proColon	Septin 9	Colorectal cancer	qMSP	Blood	Epigenomics AG	FDA/CFDA approved	USA
ICOLOCOMF	SDC2, TFPI2	Colorectal cancer	qMSP	Stool	Ammunition	Trademark registered	China
Lung EpiCheck	6-gene DNA methylation panel	Lung cancer	qMSP	Blood	Nucleix	Under clinical investigation	Israel
Lung-MeTM	SHOX2, RASSF1A	Lung cancer	qMSP	Bronchoalveolar lavage fluid	Tellgen	NMPA approved	China
GynTect	ASTN1, DLX1, ITGA4, RXFP3, SOX17, ZNF671	Cervical cancer	qMSP	Cervical scrapes	Oncgnostics GmbH	CE approved	Germany
QIAsure	FAM19A4, hsa-mir124-2 encoding gene	Cervical cancer	qMSP	Cervical scrapes	Qiagen	CE approved	Germany
ICervsure	PAX1, P16	Cervical cancer	qMSP	Cervical scrapes	Hybribio	Trademark registered	China
Bladder EpiCheck	15-gene DNA methylation (ARF, FADD, TNFRSF21, BAX, LITAF, DAPK, TMS-1, BCL2, RASSF1A, TERT, TNFRSF25, EDNRB, TBX2, TBX3, ZIC4)	Bladder cancer	qMSP	Urine	Nucleix	CE approved	Israel
PLXNA1 alteration detection kit	PLXNA1	Prostate cancer	MeFISH	Urine	Shenrui Bio Pharmaceutics	Trademark registered	China
PCDH9 alteration detection kit	PCDH9	Prostate cancer	MeFISH	Urine	Shenrui Bio Pharmaceutics	Trademark registered	China

qMSP: real-time methylation-specific PCR; MeFISH: methylation-specific fluorescence *in situ* hybridization.

All products listed are based on DNA methylation.
